# On the Hardness and Elastic Modulus of Phases in SiC-Reinforced Al Composite: Role of La and Ce Addition

**DOI:** 10.3390/ma14216287

**Published:** 2021-10-21

**Authors:** Andong Du, Lucia Lattanzi, Anders Wollmar Eric Jarfors, Jinchuan Zheng, Kaikun Wang, Gegang Yu

**Affiliations:** 1 Institute of Semi-Solid Metal Technology, China Academy of Machinery Sciences and Technology (Jiangle), No. 22 Huancheng East Road, Jiangle, Sanming 353300, China; b20170186@xs.ustb.edu.cn (A.D.); zhengjc@cam.com.cn (J.Z.); yugg@cam.com.cn (G.Y.); 2 Department of Materials Processing and Control Engineering, School of Materials Science and Engineering, University of Science and Technology Beijing, Xueyuan Road No. 30, Beijing 100083, China; 3 Department of Materials and Manufacturing, School of Engineering, Jönköping University, 551 11 Jönköping, Sweden; lucia.lattanzi@ju.se

**Keywords:** metal matrix composites, aluminium alloys, SiCp, nanoindentation, lanthanum, cerium, hardness, elastic modulus, transition metals, rare-earth elements

## Abstract

The use of silicon carbide particles (SiCp) as reinforcement in aluminium (Al)-based composites (Al/SiCp) can offer high hardness and high stiffness. The rare-earth elements like lanthanum (La) and cerium (Ce) and transition metals like nickel (Ni) and copper (Cu) were added into the matrix to form intermetallic phases; this is one way to improve the mechanical property of the composite at elevated temperatures. The α-Al_15_(Fe,Mn)_3_Si_2_, Al_20_(La,Ce)Ti_2_, and Al_11_(La,Ce)_3_, π-Al_8_FeMg_3_Si_6_ phases are formed. Nanoindentation was employed to measure the hardness and elastic modulus of the phases formed in the composite alloys. The rule of mixture was used to predict the modulus of the matrix alloys. The Halpin–Tsai model was applied to calculate the elastic modulus of the particle-reinforced composites. The transition metals (Ni and Cu) and rare-earth elements (La and Ce) determined a 5–15% increase of the elastic modulus of the matrix alloy. The SiC particles increased the elastic modulus of the matrix alloy by 10–15% in composite materials.

## 1. Introduction

The aluminium-silicon alloys (Al-Si) reinforced with oxides and carbide, commonly referred to as Al metal matrix composites (Al-MMCs), were initially investigated in the 1990s [1,2,3,4]. Interest in these materials has significantly increased in recent years [5,6,7]. The main reason behind this is the increasing demand for lightweight components, as these are the critical routes to reduce CO_2_ emission and fuel consumption [8,9]. These composites are also suitable for upcoming vehicle electrification that determines additional requirements in vehicle safety and particulate emission, especially dust from braking systems operating at 400 °C [6]. Using silicon carbide particles (SiCp) as reinforcement in Al/SiCp composites can offer high hardness and stiffness. The high hardness would promote excellent wear resistance making Al/SiCp composites highly suited for brake discs, and the increased stiffness compared to Al-Si alloys could offer a route to further weight reduction [5].

The drawback of Al-based composites lies in the high-temperature performance since the Al matrix suffers from softening during thermal exposure. Alloying elements like copper (Cu) and nickel (Ni) are specifically added to maintain good mechanical properties at high temperatures [10,11,12,13]. These elements form intermetallic compounds that are thermally stable and can withstand load bearing during temperature rise. Using too much Cu will pollute the water and ground, causing the exposure of living beings to a higher-level dose that may be harmful and lead to health problems. The aim is thus to the Cu amounts in the alloy design. [14].

Several studies focused on rare earths in the recent decade for their modifying effect on eutectic silicon [15,16]. Recently the role of lanthanum (La) and cerium (Ce) additions for the high-temperature performance of Al-based alloys became of interest [17,18,19,20,21,22,23].

In particular, Wang et al. [24] reported the formation of nanoscale (La,Ce)-based phases in an Al-Si-Mg-Zn alloy. The rapid solidification in high-pressure die-casting determines the presence of nano-precipitates in the Al matrix in the as-cast condition. The thermal stability of these phases improves the high-temperature performance and is not affected by heat treatment.

Previous work on tensile properties by Du et al. [17] reported a significant strength increase in the 200–400 °C temperature range. They concluded that the strengthening effects related to the formation of Al_11_(La,Ce)_3_ are primarily load-bearing and have modulus mismatch. Besides, the sustainability-performance-cost benefit analysis by Jarfors et al. [25] demonstrated that it is critical to consider the reinforcing effect of the alloying elements to perform an informed choice. Rare earths such as La and Ce become advantageous in terms of environmental impact and cost benefits compared to Mg.

The addition of La, Ce, and Ni and Cu into the matrix improves the mechanical property at elevated temperatures by forming intermetallic phases. Due to the hardness and modulus of phases formed not being well-studied, the present work aims to assess the hardness and elastic properties of the phases that constitute the Al/SiCp composites adapted for elevated temperatures. This investigation compared materials with and without the addition of transition metals (Ni and Cu) and rare earths (La and Ce) to the matrix alloy.

The present study aims to gain a comprehensive knowledge of the Al/SiCp composites to improve the strength at elevated temperatures to be applied in the brake disk for high-speed trains and electric vehicles. The targeted maximum service temperatures well above 420 °C. The friction and wear performance are better than the grey cast iron, and their use can reduce density by 60% and improve the thermal conductivity [5,26].

## 2. Materials and Methods

### 2.1. Material Production

In the current study, two different Al/SiCp composites denominated C0 and C1 with matrix compositions collated in Table 1, were investigated. It should be noted that La and Ce are given as nominal values due to the limitations of the analysis equipment.

The Al/SiCp materials were processed by a proprietary stir-casting method to keep porosity at a minimum level. The carbides were heat-treated at 1000 °C for one hour to grow silicon oxide (SiO_2_) on the surface of particles. The wetting angle between SiO_2_ and molten Al at that temperature will be below 68.8 degrees, leading to evenly dispersed SiC particles in the molten alloy [27]. The fraction of the reinforcing carbides was ~14 wt.%.

### 2.2. Microstructural Characterisation

Samples were mounted and prepared for metallographic observation combining the grinding steps for hard materials, SiC particles, and the polishing steps for soft materials, Al-Si matrix. Metallographic observations were performed by Olympus DSX1000 (Olympus Corporation, Shinjuku, Japan), optical microscope (OM) and JSM-7001F (JEOL, Akishima, Japan) scanning electron microscope (SEM). Octane Pro (Edax, Mahwah, NJ, USA) probe for energy X-ray dispersion spectroscopy (EDS) was employed for phase recognition.

### 2.3. Microstructure Mechanical Properties Characterisation

Nanoindentation was performed with the diamond Berkovich indenter (NanoTestTM, Micro Materials Ltd., Wrexham, UK). The tests were carried out on unetched specimens to detect the actual surface hardness. For each material, the applied load was 10 mN over a grid of 20 × 20 indentations distanced 15 µm.

The hardness *H_IT_* [GPa] come from Equation (1):

(1) $$H_{IT}=P_{max}/A_{c}$$

The *P_max_* [mN] is the peak load, and the *A_c_* [nm^2^] is the indenter contact surface projected area on the tested material.

The reduced modulus *E_r_* [GPa] come from Equation (2):

(2) $$E_{r}=\sqrt{\pi }\cdot S/2\cdot \sqrt{A_{c}}$$

In Equation (2), *S* [mN/nm] is the experimentally measured stiffness of the upper portion of the elastic unloading, which can be determined by calculating the slope of the load-displacement curve at the beginning of the unloading process. The elastic modulus *E_IT_* [GPa] was calculated from the reduced modulus *E_r_* [GPa] with Equation (3):

(3) $$E_{IT}=\left(1-v^{2}\right)/\left(\frac{1}{E_{r}}-\frac{1-v_{i}^{2}}{E_{i}}\right)$$

In Equation (3), *E_i_* = 1141 GPa is the elastic modulus of the diamond indenter, *ν_i_* = 0.07 is the Poisson’s ratio of the diamond indenter, and *ν* is the Poisson’s ratio of the tested material [28]. In the present study, *ν* = 0.18 for the SiC and *ν* = 0.3 for the other phases [29].

## 3. Results and Discussion

This section may be divided by subheadings. It should provide a concise and precise description of the experimental results, their interpretation, and the experimental conclusions that can be drawn.

### 3.1. Microstructure and Identification of Intermetallic Phases

**Figure 1:**
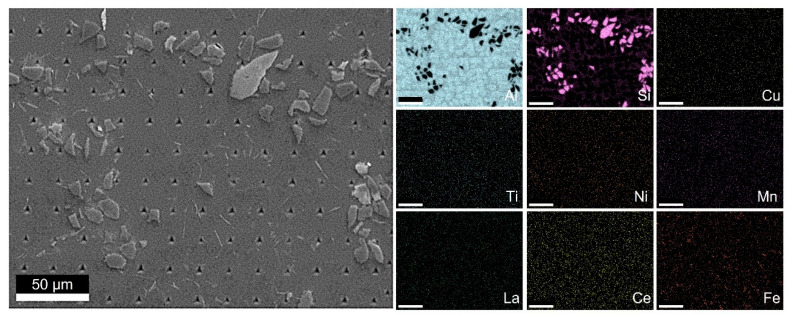
Scanning electron microscopy (SEM) image plus energy-dispersive X-ray spectroscopy (EDS) mapping: Material C0.

Figure 1 presents the SEM micrograph and related EDS element map for Material C0. The EDS data were used to identify the secondary phases formed in the matrix alloy.

**Figure 2:**
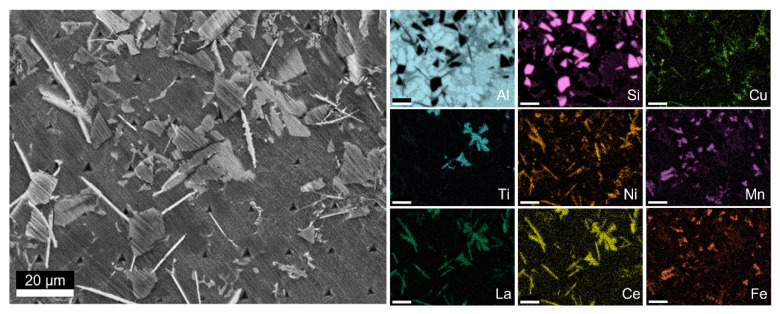
SEM image and EDS mapping: Material C1.

Figure 2 shows the SEM micrograph and related EDS element map for Material C1. In addition to the Al matrix, the eutectic Si and the SiC particles, various secondary phases were present with different morphologies.

Table 2 summarises the at.% content range of the elements in the secondary phases identified with EDS. The values given in Table 2 were compared with the atomic composition reported in the literature [30,31,32,33,34,35]. The Al content can be higher than expected from the nominal composition because of the interaction volume of the matrix with the electric beam, with an acceleration voltage of 20 kV.

The lath phase containing Fe and Mg was identified as the π-Al_8_FeMg_3_Si_6_ phase, in line with Casari et al. [30] and Ludwig et al. [31]. The excessive Al content, compared to the nominal composition, is linked to the interaction volume of the electron beam during the measurement and the size of the phase. The composition of the polygonal phase containing Fe and Mn corresponds to the α-Al_15_(Fe,Mn)_3_Si_2_ phase, with a small quantity of dissolved Ni according to Khalifa et al. [32]. The Ti(La,Ce)-based phase with polygonal morphology can be the Al_3_Ti phase with La and Ce dissolved in the lattice. The crystal structure is tetragonal with six Al atoms and two Ti atoms in each unit cell, but the at.% content of Ti does not align with the composition in Table 2. Melotti et al. [33] reported the formation of the Al_20_CeTi_2_ phase in a cast Al-MMC. The polygonal morphology and the composition in at.% align with those found in the present study (Table 2). This intermetallic has a face-centred cubic structure (*cF*184) [36], like the Al_20_LaTi_2_ phase [35,37]. Given the similar atomic structure of La and Ce, the phase in the present work can be identified as Al_20_(La,Ce)Ti_2_.

The Al(La,Ce)-based phase with lath morphology was identified as Al_11_(La,Ce)_3_ according to previous work by Du et al. [17], who reported that Cu and Ni are partially dissolved in this intermetallic phase. The presence of Ni in the Al_11_Ce_3_ phase was further confirmed by the Springer Material database [34], and it also explains the presence of Cu. The lattice structure is body-centred orthorhombic (*oI*28), and the inter-changeability of La and Ce in the Al_11_X_3_ structure was confirmed by Medvedev et al. [38].

**Figure 3:**
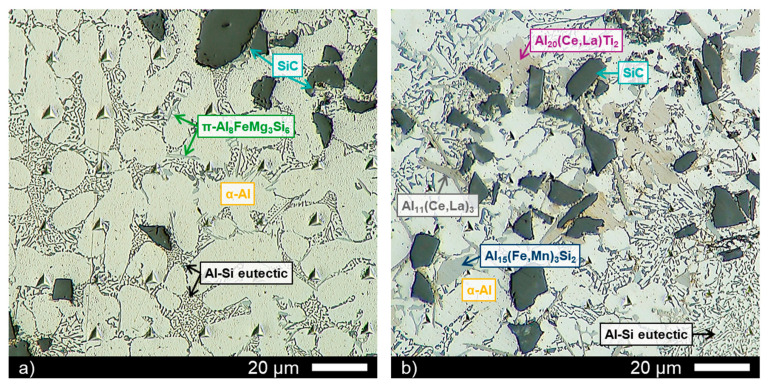
Optical microscope micrographs: (**a**) Material C0; (**b**) Material C1.

Figure 3a,b show the same regions of Figure 1 and Figure 2, respectively. The colours of the phases in the optical microscope image are different, and it helped distinguish them, as depicted in Figure 3. Figure 3a shows the microstructure of Material C0, while Figure 3b depicts the material with transition elements (Cu and Ni) and rare-earth elements (La and Ce) in the matrix alloy and the different phases listed in Table 2 showed different colours. The α-Al matrix appeared bright grey, and the black particles were the reinforcing carbides.

### 3.2. Microstructure Mechanical Properties

The displacement indentation curves included loading and unloading ramps. During the initial loading, both elastic and plastic deformation processes occurred. A slight elastic deformation recovery occurred at the unloading section, as the elastic and plastic responses of the material determined the indenter penetration depth. The hardness is correlated with the indenter penetration depth, whereas the elastic modulus is correlated with the elastic response of the phases.

**Figure 4:**
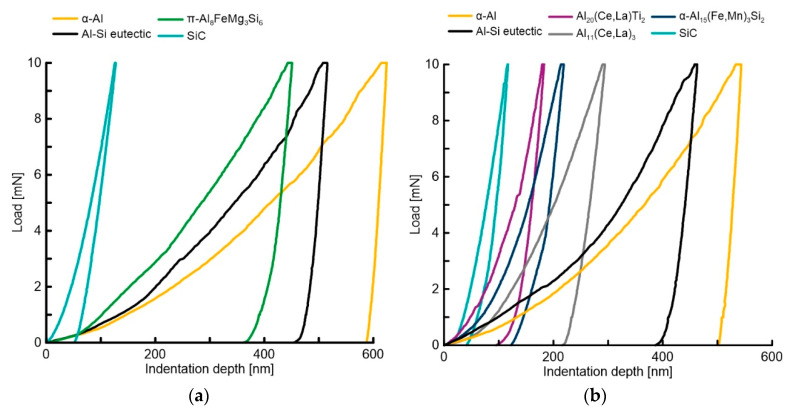
Plots of load vs. indentation depth for each phase: (**a**) Material C0; (**b**) Material C1.

The indentation curves of the two materials are shown in Figure 4. Figure 4a represents the curves of the four phases in Material C0: α-Al, SiC, Al-Si eutectic, and π-Al_8_FeMg_3_Si_6_. Figure 4b represents the curves of the six phases in Material C1: α-Al, SiC, Al-Si eutectic, α-Al_15_(Fe,Mn)_3_Si_2_, Al_20_(La,Ce)Ti_2_, and Al_11_(La,Ce)_3_. The indentation depth varied with the phases, and it was caused by the elastic and plastic response of the material. 

In Figure 4a, the α-Al curve (yellow) shows ~600 nm maximum indentation depth, and the loading ramp had a low slope, which means it was the softest phase. The maximum indentation depth of SiC (light blue curve) was ~110 nm, and the slope of the loading ramp was steep, which means it was the hardest phase. The Al-Si eutectic (black curve) and the π-Al_8_FeMg_3_Si_6_ (green curve) phases presented a maximum indentation depth of ~500 nm and ~420 nm. The higher hardness of SiC phases shown short indentation depth, and the soft α-Al matrix had the deepest indentation depth. The sequence of indentation depth corresponded opposite to the hardness of the phases.

Figure 4b shows the curves of the α-Al_15_(Fe,Mn)_3_Si_2_ (blue curve) phase and the (La,Ce)-based phases, Al_20_(La,Ce)Ti_2_ (purple curve) and Al_11_(La,Ce)_3_ (grey curve). At the loading curve of the SiC, Al_20_(La,Ce)Ti_2_, Al-Si eutectic and α-Al phases shown a discontinue so-called “pop-in” effect, which could be explained by dislocation activities [39]. The displacement changes at the load are related to the tested material’s maximum shear strength, and it is linked to the nucleation and propagation of dislocation.

Table 3 lists the hardness of the phases in materials C0 and C1. The hardness of α-Al in C0 and C1 composites are 0.98 GPa and 1.24 GPa, respectively, and the pure Al is 0.7 GPa [40,41]. The hardness of α-Al in C1 material is slightly higher than the C0 alloy. This value is in line with previous studies by Nayak et al. [42] and Youn et al. [43], who investigated Al-7Si alloys with 0.1–0.4 wt.% of Mg. The increase in hardness of the α-Al is due to the strengthening effect of the solid solution of Si, Cu and Mg in the present study. This effect was confirmed by comparing the values of 1.45–1.55 GPa reported by Tupaj et al. [44] and Chen et al. [40,41]. Both studies investigated hyper-eutectic Al–Si alloys with high contents of Cu and Mg in the 1.4–2 wt.% and 0.9–1.33 wt.% ranges, respectively. In line with what was observed for the α-Al, the hardness value of the Al–Si eutectic is 1.51 ± 0.04 GPa in Material C0, and it is 2.00 ± 0.13 GPa in Material C1.

Moving to the intermetallic phases, the π-Al_8_FeMg_3_Si_6_ phase in Material C0 showed a hardness value of 2.14 ± 0.56 GPa, which contrasts with the Vickers hardness of 6.85 GPa reported by Farkoosh et al. [45] from a non-clarified reference. The direct comparison between Berkovich and Vickers indentations can be performed only after careful conversion [46]. In Material C1, several intermetallic phases were detected, and the α-Al_15_(Fe,Mn)_3_Si_2_ showed a hardness of 8.44 ± 3.04 GPa. Once again, this value is lower than the data available in the literature. Tupaj et al. [44] reported ~14.7 ± 2.0 GPa for the Al(Fe,Mn)Si phase supersaturated with Cr and V, while Chen et al. [40,41] reported 10.8 ± 0.3 GPa. They also observed that hardness was constant with increasing concentrations of Fe and Mn, from 14.21 to 23.13 at.% of Fe+Mn.

The hardness of the Al_20_(La,Ce)Ti_2_ phase was 6.78 ± 0.76 GPa and the Al_11_(La,Ce)_3_ showed 2.82 ± 0.61 GPa. For the former phase, Ma et al. [35] calculated hardness (H) by a semi-empirical relation in Equation (4) that combines bulk (*B*) and shear (*G*) moduli [GPa]:

(4) $$H=0.92G^{0.708}{\left(B/G\right)}^{1.137}$$

The resulting hardness values were 12.2 GPa for both Al_20_CeTi_2_ and Al_20_LaTi_2_, and this value is almost double the hardness measured in the present work. The authors derived the Al hardness with the same Equation (4) resulting in 2.87 GPa, a value double than the ones measured in the present work.

The hardness of SiC was in the range 27.7–30.3 GPa, and this was the hardest phase in the matrix. Therefore, the SiC particle could play a crucial role in protecting the matrix from wear damage.

Table 3 shows the elastic modulus of the different phases in Materials C0 and C1. The elastic modulus of newly formed phases in C1 alloy are Al_20_(Ce,La)Ti_2_, Al_11_(La,Ce)_3_ and α-Al_15_(Fe,Mn)_3_Si_2_, their elastic modulus are 148.1 ± 13.6 GPa, 124.3 ± 27.4 GPa, and 158.0 ± 32.8 GPa, respectively. The elastic modulus of all three phases is higher than the phases in the C0 alloy, except the carbide.

Wang et al. [47] reported a modulus of 78.6–84.1 GPa for pure Al. In the present work, the modulus was 88.2 ± 1.5 GPa in material C0 and 93.9 ± 3.4 GPa in material C1, with a difference of 6%. For the Al_20_(Ce,La)Ti_2_ phase, Ma et al. [35] reported a Poisson’s ratio of 0.2, an elastic modulus of 141–146 GPa. Updating the Poisson’s value in Equation (1), the resulting elastic modulus is 156 ± 14 MPa instead of 163 ± 14 MPa. Similar to what was observed for hardness, the elastic modulus of phases in C1 alloy is higher than the C0 alloy, which means the transition metals (Ni and Cu) and rare earth (La and Ce) contribute to the improvement of elastic modulus.

The design of automotive structure components made from Al alloys is usually based on requirements about yield strength and elastic modulus [48]. The aluminium’s strength usually meets the requirement, while the elastic modulus usually does not. Introducing secondary phases with high modulus could improve the overall elastic modulus of the material.

The overall modulus of material usually depends on the volume fraction, modulus, size, distribution, and the interface between the secondary phases and matrix. Due to the difference of elastic modulus between particle and matrix, plastic deformation easily occurs mainly at the interface region and thus induces the increased density of dislocation. The increased elastic modulus of the phases contribute to the matrix of stresses [49]. It also improves the elastic modulus value of the matrix and can be estimated by theoretical modelling. The most frequently used mathematical model is the Halpin–Tsai model for particle-reinforced composites [50], expressed in Equation (5).

(5) $$E_{c}=\frac{E_{m}\left(1+2sqV_{p}\right)}{1-qV_{p}}$$

In Equation (5), *E_c_* and *E_m_* [GPa] are the composite and matrix elastic moduli, respectively. The parameter *s* is the aspect ratio of the reinforcing particles, *V_p_* [%] is volume fraction, *q* is a geometrical parameter estimated by Equation (6).

(6) q=(EpEm)−1(EpEm)+2s

In Equation (6), *E_p_* [GPa] is the elastic modulus of the reinforcing particles.

Equations (5) and (6) enable the calculation of SiC particle’s contribution to the composite material’s elastic modulus. The aspect ratio of SiC particles resulted in *s* ~2, and their volume fraction was *V_p_* ~14% in both cases. The elastic modulus of SiC in materials C0 and C1 were 335.2 GPa and 402.5 GPa, respectively, according to Table 3.

The rule of mixture was used to predict the modulus of the matrix alloys for materials C0 and C1. For reinforced composites, the elastic modulus *E_m_* of the composites can be calculated by Equations (7) and (8) [51]. Equation (7) is the upper-bound modulus and assumes that the reinforcing phases are parallel to the load direction and Equation (8) is representative of the lower-bound modulus, with the reinforced phases transverse to the load direction.

(7) $$E_{m}=fE_{f}+\left(1-f\right)E_{a}$$

(8) $$E_{m}={\left(\frac{f}{E_{f}}+\frac{1-f}{E_{a}}\right)}^{-1}$$

*E_f_* is the elastic modulus of phases, *E_a_* is the elastic modulus of the α-Al, *f* is the volume fraction of the reinforcing phases. Thermocalc software was used to calculate the volume fraction of the phases in the matrix alloys of C0 and C1, and the result are listed in Table 4. The matrix alloys can be seen as composite materials reinforced by the secondary phases, and thus their elastic moduli (Table 3) were used in Equations (7) and (8) to calculate the matrix moduli listed in Table 5. 

Table 5 lists the elastic moduli of the matrix alloys calculated with the upper and lower bound of the rule of mixture, defined in Equations (7) and (8). These values were used in the Halpin–Tsai model, expressed in Equation (5), to describe the composite materials.

As listed in Table 5, The *E_m_* of the matrix alloys C0 and C1 are 87.7–90.5 GPa and 93.4–105.1 GPa, respectively. The transition metals (Ni and Cu) and rare-earth elements (La and Ce) determined a 5–15% increase of the elastic modulus of the matrix alloy. After calculation with Equations (5) and (6), the elastic modulus of the composites Ec resulted in 111.0–113.9 GPa for material C0 and 120.9–133.1 GPa for material C1. This result highlights that the SiC particles increased the elastic modulus of the matrix by 10–15% in both composite materials. Equation (9) from Ceschini et al. [49] describes the strength contribution of the modulus mismatch:

(9) $$C_{MM}\approx 3\sqrt{2}\cdot \beta \cdot \sqrt{b}\cdot G_{m}$$

*C_MM_* resulted 1.47 MPa·from previous work [17]. With *β* = 0.5 and the Burgers vector *b* = 0.286 nm for in Al, the resulting shear modulus of the matrix is *G_m_* = 41 GPa. This in turn corresponds to an elastic modulus of 107 GPa corresponding to *E_m_*. In the current work the upper bound estimate for *E_m_* was 105.1 GPa for material C1, validating the rules of mixture usage as it is reasonable. This correspondence also confirms a significant dispersion hardening effect, strengthening the matrix to better match the SiC reinforcement in a brake disc application.

## 4. Conclusions

The present study investigated the influence of transition metals (Cu and Ni) and rare-earth elements (La and Ce) addition to the microstructure of an Al/SiCp composite. The hardness and elastic modulus of the secondary phases were measured by nanoindentation. The following conclusions can be drawn:

- The addition of La and Ce formed the α-Al_15_(Fe,Mn)_3_Si_2_, Al_20_(La,Ce)Ti_2_, and Al_11_(La,Ce)_3_ phases, and the transitions metals were dissolved in these intermetallic phases.
- The hardness and elastic modulus of the phases of Al_11_(La, Ce)_3_ are 2.8 ± 0.6 GPa and 124.3 ± 27.4 GPa, respectively; Al_20_(Ce,La)Ti_2_ has hardness 6.78 ± 0.78 GPa and elastic modulus 148.1 ± 13.6 GPa; the α-Al_15_(Fe,Mn)_3_Si_2_ phase has hardness 8.44 ± 3.04 GPa and elastic modulus 158.0 ± 32.8 GPa; π-Al_8_FeMg_3_Si_6_ has hardness 2.1 ± 0.6 GPa and elastic modulus 111.0 ± 44.0 GPa.
- Based on the rule-of-mixture, the calculate elastic modulus of the matrix alloys C0 and C1 are 87.7–90.5 GPa and 93.4–105.1 GPa.
- Based on the Halpin–Tsai model for particle-reinforced composites, the calculated elastic modulus ranges of C0 and C1 composite materials are 111.0–113.9 GPa and 120.9–133.1 GPa, respectively. The SiC particles increased the elastic modulus of the matrix by 10–15% in both composite materials.

## Figures and Tables

**Table 1:** Chemical composition of matrix alloys [wt.%].

Matrix Alloy	Si	Cu	Ni	Fe	Mn	Ti	Mg	Ce	La	Al
C0	10	0.2	-	0.1	-	0.1	0.8	-	-	bal.
C1	10	1.9	1.9	0.1	0.8	0.1	0.8	1	1	bal.

**Table 2:** Chemical composition range [at.%] of intermetallic phases from EDS spectra.

Assigned Phase/ Morphology	Composition	Al	Si	Fe	Mg	Mn	La	Ce	Ti	Ni	Cu	Material
π-Al_8_FeMg_3_Si_6_/ lath	detected	72.5–75.3	14.4–17.9	1.2–2.1	7.6–9.2	-	-	-	-	-	-	C0
	nominal	44.4	33.3	5.5	16	-	-	-	-	-	-	
α-Al_15_(Fe,Mn)_3_Si_2_/ polygonal	detected	70.0–71.6	10.0–11.8	3.5–3.54	-	11.7–12.0	-	-	-	1.47–1.5	-	C1
	nominal	65.2	8.6	13	-	13	-	-	-	-	-	
Al_20_(Ce,La)Ti_2_/ polygonal	detected	82.1–83.6	3.8–6.1	-	-	-	1.9–2.1	2.5–2.7	6.8–7.2	-	0.69–0.74	C1
	nominal	83.3	–	-	-	-	4.1	4.1	8.2	-	-	
Al_11_(Ce,La)_3_/ lath	detected	58.0–65.9	15.8–18.1	-	-	-	1.0–4.8	0.8–4.0	-	3–12.2	1.6–6.6	C1
	nominal	64.7	-	-	-	-	17	17	-	-	-	

**Table 3:** Hardness and elastic modulus of phases formed in materials C0 and C1.

Phase	Composite	Hardness/GPa	Elastic Modulus/GPa
α-Al	C0	0.98 ± 0.01	88.2 ± 1.5
	C1	1.24 ± 0.05	93.9 ± 3.4
Al-Si eutectic	C0	1.51 ± 0.04	97.2 ± 3.2
	C1	2.00 ± 0.13	106.4 ± 5.1
SiC	C0	27.7 ± 2.6	335.2 ± 30.7
	C1	30.2 ± 2.3	402.5 ± 45.0
π-Al_8_FeMg_3_Si_6_	C0	2.1 ± 0.6	111.0 ± 44.0
Al_20_(Ce,La)Ti_2_	C1	6.8 ± 0.8	148.1 ± 13.6
Al11(La,Ce)3	C1	2.8 ± 0.6	124.3 ± 27.4
α-Al_15_(Fe,Mn)_3_Si_2_	C1	8.4 ± 3.0	158.0 ± 32.8

**Table 4:** Fraction of phases, calculated by Thermocalc, formed in the matrix alloys of materials C0 and C1.

Phase	Composite	Fraction
α-Al	C0	0.89
	C1	0.76
Al-Si eutectic	C0	0.09
	C1	0.08
SiC	C0	0.14
	C1	0.14
π-Al_8_FeMg_3_Si_6_	C0	0.004
Al_20_(Ce,La)Ti_2_	C1	0.030
Al_11_(La,Ce)_3_	C1	0.037
α-Al_15_(Fe,Mn)_3_Si_2_	C1	0.028

**Table 5:** Calculated elastic moduli of matrix alloys and composites C0 and C1, with and without SiC_p_. The rules of mixtures were used for the matrix alloys, and the Halpin–Tsai model was used for the composite materials.

Composite	Rules of Mixture	Em [GPa]	Ec [GPa]
C0	upper-bound modulus equation	90.5	113.9
	lower-bound modulus equation	87.7	111.0
C1	upper-bound modulus equation	105.1	133.1
	lower-bound modulus equation	93.4	120.9

## Data Availability

Data available in a publicly accessible repository.
